# Greater Tuberosity Healing Rate and Clinical Results Following RSA Are Similar for Two Fracture-Specific Implant Systems

**DOI:** 10.3390/jcm13226967

**Published:** 2024-11-19

**Authors:** Dani Rotman, Omer Avraham, Yariv Goldstein, Efi Kazum, Jorge Rojas Lievano, Ofir Chechik, Eran Maman

**Affiliations:** 1 Division of Orthopedic Surgery, Tel Aviv Sourasky Medical Center, Faculty of Medicine, Tel Aviv University, Tel Aviv 6997801, Israel; 2 Orthopedic Surgery Department, Laniado Hospital, Netanya 4244916, Israel; 3 Adelson Faculty of Medicine, Ariel University, Ariel 4070000, Israel; 4 Orthopedic Department, Assuta Ashdod Medical Center, Ashdod 7747629, Israel; 5 Beer Sheva Faculty of Medicine, Beer Sheva University, Beer-Sheva 8410501, Israel; 6 Department of Orthopedics and Traumatology, Hospital Universitario Fundación Santa Fe de Bogotá, Bogotá 110111, Colombia; 7 School of Medicine, Universidad de Los Andes, Bogotá 110111, Colombia

**Keywords:** proximal humerus fracture, reverse shoulder arthroplasty, greater tuberosity, tuberosity healing, bone cement, cementless, fracture-specific implant

## Abstract

**Background:** Various fracture-specific reverse shoulder arthroplasty (RSA) systems exist on the market. We set out to examine whether the type of prosthesis used and the means of fixation (cemented or non-cemented) influenced the rate of tuberosity healing or the functional outcome of the operation. **Methods:** This retrospective cohort multicenter study included 146 patients who underwent RSA for an acute three- or four-part proximal humerus fracture and had a minimum follow-up of one year. Six fellowship-trained surgeons at two different centers performed all operations. The implants were either Tornier Aequalis or Depuy Delta Xtend, both fracture-specific Grammont-style systems. **Results:** The mean age ± standard deviation (SD) was 76 ± 7 years, and 83% of patients were female. The mean ± SD follow-up time was 30 ± 31 months. The Aequalis prosthesis was used in 82 patients (56%), and the Delta Xtend in 64 patients (44%). A total of 105 RSAs (72%) were cemented. Tuberosity healing rate was similar for the two implant systems (71% Aequalis vs. 82% Delta Xtend, *p* = 0.15) and for the cemented or non-cemented, respectively (73% cemented vs. 83% non-cemented, *p* = 0.22). There was no significant difference in the motion and functional outcomes between the two implant systems in this study. **Conclusions:** RSA for complex PHF in the elderly has similar short-term results, regardless of the type of fracture-specific implant or the fixation technique (cemented vs. cementless).

## 1. Introduction

Reverse shoulder arthroplasty (RSA) has become popular in the last two decades in the management of patients who have fractures of the proximal humerus with associated severe osteoporosis and RC dysfunction [1]. It has the advantages of a better subjective functional score, forward flexion, and lower chances of revision surgery than osteosynthesis [2] and hemiarthroplasty [3,4].

As in hemiarthroplasty, the correct repositioning and healing of the tuberosities were suggested to positively influence the range of movements and functional outcomes [5]. Similarly, the use of cementless fixation was postulated as a possible method to increase the rate of tuberosity healing [6], as the presence of cement in the area of the tuberosities was shown in hemiarthroplasty to decrease the rate of tuberosity healing [7].

In recent years, several “fracture-specific” implant systems have been introduced, with features claiming to allow better tuberosity healing [8]. Specifically, the Equalis Fx (Tornier Wright Medical, Montbonnot, France) has a metaphyseal window for bone grafting and hydroxyapatite coating, whereas the Delta Xtend Fx (DePuy Synthes, Warsaw, IN, USA) has a collar designed for tying the tuberosities to the implant, porous coating, and the option of cementless implantation.

This multicenter retrospective study aims to compare these two popular Grammont-style fracture-specific implant systems and determine whether the type of prosthesis used and the means of fixation (cemented or non-cemented) influence the radiographic greater tuberosity healing rate or the functional outcome of this operation. Our hypotheses were as follows: (1) the Delta Xtend prosthesis would achieve better tuberosity healing due to the collar design allowing for more straightforward tuberosity reduction and fixation; (2) cementless implantation would result in higher rates of tuberosity healing. Such differences, if found, should have implications on surgeon preferences and the process of informed consent for RTSA [9,10].

## 2. Materials and Methods

**Study population.** This was a retrospective cohort multicenter study. The study cohort included all patients who underwent RSA for an acute three- or four-part proximal humerus fracture in one of two urban medical centers between January 2013 and June 2021. The exclusion criteria included the following: (1) implant used other than Tornier Aequalis Fx or Depuy Delta Xtend; (2) radiographic follow-up of less than 4 months (sufficient to assess tuberosity union) or clinical follow-up of less than one year. Patients with clinical follow-up of less than one year were included in the radiographic results analysis but were excluded from the clinical results analysis.

The original cohort included 170 patients, of whom 13 were excluded due to another implant being used and 11 due to insufficient follow-up, leaving 146 patients in the study.

**Clinical and radiologic assessments.** Clinical evaluation included active range of motion, pain score on a numeric rating scale (NRS) from 0 to 10; subjective shoulder value (SSV) [11]; and a quick disabilities of the arm and shoulder score (Q-DASH) [12]. Active ROMs comprised forward flexion, abduction, external rotation with the elbow at the side, and internal rotation, which were reported using Constant score levels [13] (lateral thigh, buttock, lumbosacral junction, T12, inferior tip of scapula, interscapular region).

**Figure 1:**
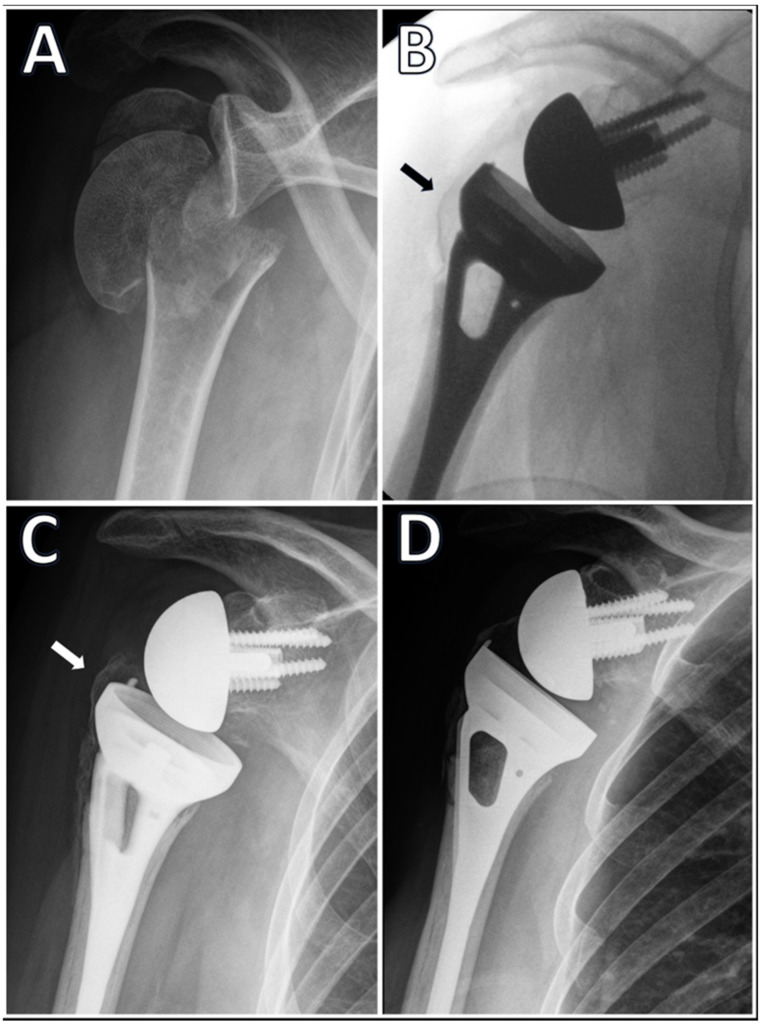
A 73-year-old female presented with a 3-part displaced fracture of the proximal humerus (**A**); she underwent RSA using Tornier Aequalis reversed fracture prosthesis. Intraoperative fluoroscopy shows a good reduction in the greater tuberosity (black arrow) (**B**); an AP radiograph taken 6 weeks after surgery shows the proximal migration of the greater tuberosity (white arrow), which now overhangs the tray of the prosthesis, and is not in continuity with the shaft (**C**); at 3.5 months, the greater tuberosity is almost completely absorbed and is not considered united (**D**).

Radiologic assessments included intraoperative C-arm imaging to determine sufficient reduction and plain radiographs during follow-up to assess tuberosity migration and healing. AP views with the arm in internal and external rotation were routinely performed. Sufficient reduction in the greater tuberosity was defined as the tip of the tuberosity not hanging more than 5 mm over the upper rim of the implant in the intraoperative radiographs [14]. Migration was defined as a proximal migration of more than 5 mm of the greater tuberosity from its original postoperative location (Figure 1). The healing of the tuberosity was defined as the identification of the tuberosity on a plain anteroposterior (AP) radiograph in continuation with the humeral shaft. All radiographs were independently assessed for this study by two fellowship-trained shoulder surgeons who were not involved in the original treatment of the patients (DR and EK). In cases of disagreement, the two raters later discussed the cases together until reaching a consensus.

**Surgical procedures.** All surgeries were performed by one of six fellowship-trained shoulder surgeons. The patients were put in a beach chair or lazy beach chair position under general anesthesia with an interscalene block. A deltopectoral approach was utilized in all cases. The long head of the biceps tendon was commonly used as a mark for the border between the lesser and greater tuberosities, and these were tagged by sutures, followed by the removal of the humeral head. Following preparation of the glenoid and humeral shaft and the implantation of a standard (not lateralized) glenosphere, two holes were drilled at the anterior and lateral cortex of the diaphysis 2 cm distal to the fracture site, and three nonabsorbable sutures were passed through them. The humeral stem was inserted in 20° retroversion. In all cases implanting Tornier Aequalis, bone cement was used for humeral fixation, whereas in cases implanting Depuy Delta Xtend, bone cement was used at the surgeon’s discretion. Following reduction using a polyethylene component fitted to achieve optimal deltoid tension, the greater and lesser tuberosities were secured in their anatomic positions. Suture management techniques varied among surgeons, but all included tying the tuberosities to one another and securing them to the shaft using the three sutures passed before implanting. Tying the tuberosities to the implant was commonly, but not uniformly, carried out.

**Postoperative rehabilitation and follow-up.** Follow-up visits, which included clinical assessment and radiographs, were scheduled at 2, 6, and 12 weeks following surgery, and later at 6 months, one year, and yearly afterward. Postoperatively, the arm was kept in a sling for 6 weeks, and during this period, pendulum and self-assisted passive exercises were encouraged. Thereafter, self-assisted active range of motion exercises were started, gradually introducing strengthening exercises until all restrictions were lifted at 3 months.

**Statistical Analysis.** The data were summarized using means and standard deviations for continuous variables and counts and percentages for categorical variables. The Comparison of healing rates and the clinical and functional outcomes between subgroups was performed using Chi-square and *t*-tests, respectively. All statistical tests were 2-sided, and significance was set at alpha = 0.05. Analyses were performed using Stata 14 (StataCorp. 2015. Stata Statistical Software: Release 14. College Station, TX, USA: StataCorp LP.)

## 3. Results

The final study group included 146 patients. The mean ± standard deviation (SD) age was 76 ± 7 years, and 83% of patients were women. The mean ± SD in-clinical follow-up time was 30 ± 31 months, and the mean ± SD phone follow-up time was 63 ± 29 months.

The Aequalis Reversed Fracture prosthesis was used in 82 patients (56%, mean age 77.3, 82% female), and the Delta Xtend Reverse Shoulder System was used in 64 patients (44%, mean age 75.0, 84% female). A total of 105 RSAs (72%, mean age 76.9, 82% female) were cemented, and the remaining 41 RSAs (28%, mean age 74.6, 85% female) were implanted without cement.

**Figure 2:**
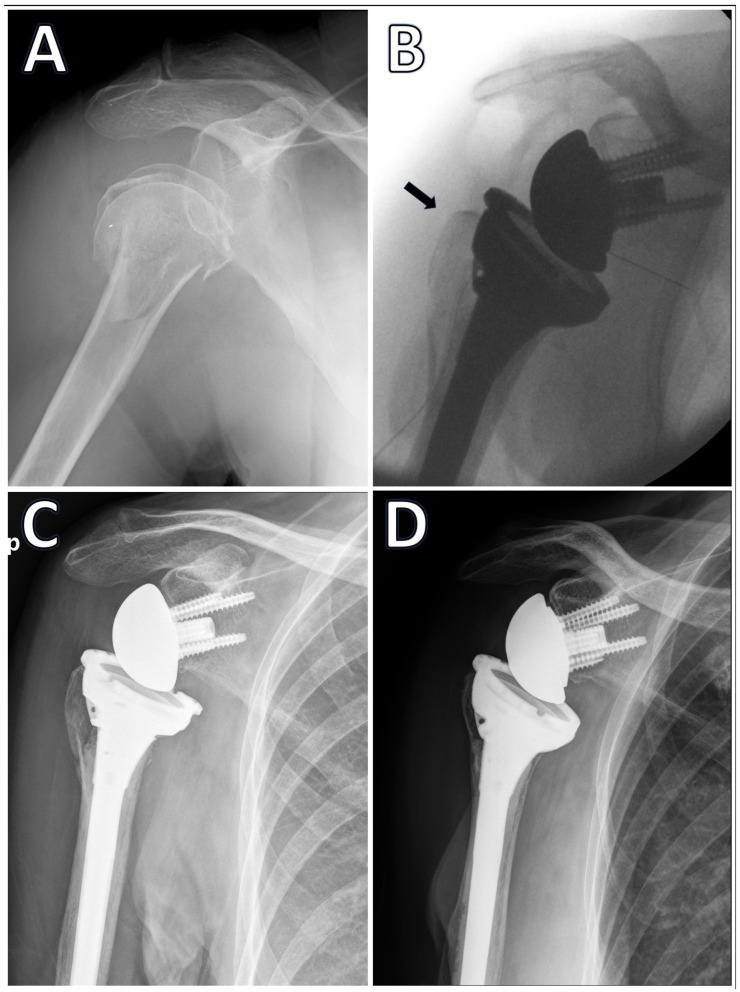
A 77-year-old female presented with a 4-part displaced fracture of the proximal humerus (**A**); she underwent RSA using a Depuy Delta Xtend shoulder system, which was implanted with bone cement. Intraoperative fluoroscopy shows good reduction in the greater tuberosity (black arrow) (**B**); an AP radiograph at 4 weeks after surgery shows no displacement of the greater tuberosity (**C**); at 15 months after surgery, there is a complete healing of the greater tuberosity in continuity with the humeral shaft (**D**).

The greater tuberosity healing rate was 76%. The tuberosity healing rate was similar for the two RSA implant systems used in this study (71% Aequalis vs. 82% Delta Xtend, *p* = 0.15). Similarly, tuberosity healing was similar for cemented versus non-cemented implants when examining all implants (73% cemented vs. 83% non-cemented, *p* = 0.22) and for Delta Xtend only (78% vs. 87%, *p* = 0.21). Tuberosity migration was noted in 20 patients (14%), with no significant difference between Aequalis (12%) and Delta Xtend (16%, *p* = 0.55). Detailed clinical and functional outcomes for the two RSA implant systems are presented in Table 1. The cases of the failed and successful healing of the greater tuberosity are presented in Figure 1 and Figure 2, respectively.

The range of motion data were available for 133 patients. Active forward flexion, abduction, and external rotation with the arm by the side were 117° ± 37°, 111°± 31°, and 14° ± 17°, respectively. The median internal rotation score was 3 points (sacrum). There was no significant difference in the active forward flexion, abduction, external rotation, or internal rotation between the two implant systems in this study (Table 1).

Functional scores were evaluated using the Q-DASH, SSV, and pain NRS scores. At final follow-up, the mean Q-DASH score was 41.1 ± 24.4, the mean SSV score was 64.8 ± 23.8, and the pain NRS was 3.1 ± 2.9. There were no significant differences in any of the functional scores between the two implant systems utilized in this study (Table 1).

## 4. Discussion

This study found that the rate of greater tuberosity healing following RSA for complex proximal humerus fracture in elderly patients was not significantly affected by the choice of fracture-specific implant system, nor by fixation type (cemented or uncemented). Similarly, there was no significant difference between the clinical outcomes of patients treated by either implant. As these results were generally good, this is yet another example of the robustness of RSA in treating these patients. Nonetheless, a trend in a higher rate of tuberosity healing in cementless fixation and with the Delta Xtend system may prove significant in a larger study.

The current literature comparing traditional and fracture-specific implants is scarce and conflicting: In the largest study thus far, Claro et al. retrospectively examined 112 cases, of which 86 were treated with Equalis Fx stem and 26 with Equalis II (not a fracture-specific stem). They found that the fracture-specific stem was associated with higher rates of anatomical tuberosity healing and achieving a functional range of motion [15]. In contrast, Imiolczyk et al. [16] and Jeong et al. [17] retrospectively compared the results of 26 and 45 cases treated with one of the prostheses mentioned above and found no significant difference in the radiographic or clinical results.

This is the first study to compare the results of two popular fracture-specific implants. These implants were explicitly designed for the treatment of proximal humerus fractures but exhibit different features to promote tuberosity reduction and healing [8].

The Equalis Fx system has (1) a metaphyseal window to accommodate a bone graft harvested from the humeral head. This bone graft is said to improve the prosthesis integration to the humerus and the tuberosities. Surgeons often also use this window to tie the tuberosities to the prosthesis, thus stabilizing their fixation. (2) The Equalis Fx system also has a low profile metaphyseal design to facilitate anatomical reduction in the greater tuberosity and a (3) hydroxyapatite coating that improves implant integration to bone by its osteoconductive properties and enhanced bone ingrowth.

The Delta Xtend Fx has (1) a suture collar that is claimed to reduce the chance of malposition and healing of the tuberosities by both acting as a mechanical buttress to block proximal migration of the tuberosities, and by having multiple holes for suture placement; (2) a porous coating that improves implant integration by providing a suitable structure for bone cells to proliferate and grow into the implant, creating a mechanical interlock; and (3) the option of cementless implantation, as cementing of the prosthesis is thought to reduce the chances of tuberosity healing (see discussion later).

Our results indicate that none of these two prostheses is substantially superior to the other in promoting tuberosity healing. Notwithstanding, as each design has several different features, and as many other variables affecting tuberosity healing were not assessed in this study, we cannot separately assess these design features for their contribution to tuberosity healing.

The reported rate of radiographic tuberosity healing in RSA is 65–85% [5,18,19]. Our results of 75% healing rate are well within this standard. Tuberosity healing is considered an important step in achieving better clinical outcomes [5]. Jain et al. performed a meta-analysis to assess the effect of tuberosity healing after RSA. This analysis included 382 cases and found a significant difference in ROM between healed GT and non-healed GT cases (active forward flexion 134.1° vs. 112.5°, abduction 114.8° vs. 95.1°, external rotation 27.8° vs. 7.6°). However, only a modest difference was noted in the functional score (Constant score 63.5 vs. 56.6). In a more recent meta-analysis, Buchman et al. examined a total of 376 patients, of which 295 (78.5%) demonstrated healing of the GT whereas 81 (21.5%) did not. The healed GT cohort exhibited a better postoperative range of motion: forward flexion was improved by 24.1° [95% CI 14.7–33.6], external rotation by 15.3° [95% CI 10.2–20.4], and internal rotation by 0.7 [95% CI 0.2–1.2]. The Constant score was also better in the healed GT cohort by 9.9 [95% CI 2.9–16.9], whereas differences in the ASES score, subjective shoulder value (SSV), and pain were insignificant. [19]. The authors concluded that the healing of the greater tuberosity after reverse shoulder arthroplasty for proximal humerus fracture yields improved the postoperative range of motion and strength, while the patient-reported outcomes (subjective function and pain) are not significantly affected. This indicates the merit of RSA as a treatment option for PHF, regardless of the likelihood of the GT healing [19].

Cementless stems are considered a possible way to increase tuberosity healing. Our cohort saw a trend towards a higher rate of tuberosity healing with non-cemented implants. However, this did not reach statistical significance (73% cemented vs. 83% non-cemented, *p* = 0.22 for all implants, and 78% vs. 87%, *p* = 0.21 for Delta Xtend only). Singh et al., examining 84 cases of shoulder hemiarthroplasty, reported that patients with anatomic healing of the greater tuberosity had cement near or under the tuberosities 32% of the time, whereas patients with nonunion or resorption had cement near the tuberosities 66% of the time (*p* = 0.002). They concluded that cementation should be kept at a minimum of 5 mm below the level of the tuberosity fracture [7]. On the contrary, Rossi et al. performed a meta-analysis pooling 34 studies reporting results of cemented and uncemented RSA stems. The overall rate of tuberosity healing was 72.5% (range, 64.9–80.1%), and there was no significant difference between the cemented (70.9% [95% CI 61.4–80.4%]) and uncemented (78.0% [95% CI 71.6–84.3%], *p* > 0.05) [6]. Interestingly, these pooled results are similar to ours, with a small but insignificant trend towards better tuberosity healing with uncemented stems. While these published results do not reach statistical significance, they consistently show a trend in higher healing rates of 5–10% with cementless stems. Better-powered studies might turn this difference into a statistically significant one, but the clinical significance would also have to be examined.

Another concern about using uncemented stems is the possible loss of proximal bone due to stress shielding [20], which is more common in elderly patients [21]. While stress shielding does not affect clinical results in the short term [21], it may have implications if revision surgery is needed. Nonetheless, stress shielding was not examined in this study.

The main limitations of this study are as follows: (1) the retrospective nature of the study and the lack of randomization introduce a possible selection bias. For example, some surgeons prefer one of the two implant systems; (2) only the healing of the greater tuberosity was assessed. The healing of the lesser tuberosity could not reliably be assessed using the standard radiographs on hand. The healing of the lesser tuberosity should have implications on the internal rotation and possibly other outcomes of this study; (3) radiographic tuberosity healing may be inadequate to assess the function of the shoulder external rotators, i.e., there is probably a functional difference between patients whose GT held in its place but dissolved to patients whose GT was displaced after surgery and dissolved in malposition. These nuances were not assessed in this study. (4) While we compared two different implant systems, only one could be implanted without cement (Delta Xtend), so the results of the cementless stems come from this implant only. This reduces the generalizability of our results regarding the effect of cementation, as they come from only one implant and are probably also influenced by its design.

## 5. Conclusions

The radiographic greater tuberosity healing rate and the clinical results following revere total shoulder arthroplasty for complex proximal humerus fracture in the elderly were almost similar for the two fracture-specific implant systems examined, regardless of the fixation technique (cemented vs. cementless). The choice of implant system and fixation type can rely more on surgeon preference and possible future considerations, as the short-term clinical and radiographic results are generally good.

## Figures and Tables

**Table 1:** Comparison of average range of motion and functional outcomes between the two RSA implant systems.

	Tuberosity Healing (%)	Forward Flexion	Abduction	ER at Side	IR at Back	Q-DASH	SSV	Pain NRS
Aequalis	71%	113 ± 37.5	108 ± 37.9	14.2 ± 18.0	Sacrum	42.6 ± 24.6	62.9 ± 21.9	2.7 ± 2.8
Delta Xtend	82%	122 ± 35.7	114 ± 40.4	14.1 ± 15.1	Sacrum	39.1 ± 24.3	66.8 ± 25.6	3.5 ± 2.9
*p*-value	0.147	0.137	0.418	0.945	1.00	0.489	0.381	0.09

Data are presented as mean and standard deviation unless otherwise indicated. ER, external rotation; IR, internal rotation (median vertebral level); Q-DAHS, Quick Disabilities of the Arm Shoulder and Hand; SSV, Subjective Shoulder Value; NRS, Numerical Rating Scale.

## Data Availability

The data presented in this study are available on request from the corresponding author.
